# Sex, Perceived Social Support, and Physical Activity as Correlates of Depressive Symptoms in Informal Caregivers: A Cross-Sectional Analysis of the Spanish National Health Survey 2023

**DOI:** 10.3390/healthcare14203485

**Published:** 2026-10-16

**Authors:** Sabina Barrios-Fernandez, Diana Salas-Gómez, Cristina Mendoza-Holgado, Angel Denche-Zamorano

**Affiliations:** 1 Social Impact and Innovation in Health (InHEALTH), Faculty of Nursing and Occupational Therapy, University of Extremadura, 10003 Cáceres, Spain; sabinabarrios@unex.es; 2 Social Impact and Innovation in Health (InHEALTH), Faculty of Sports Sciences, University of Extremadura, 10003 Cáceres, Spain; denchezamorano@unex.es

**Keywords:** informal care, exercise, social support, mental disorders, health survey, women’s health

## Abstract

**Highlights:**

**What are the main findings?**

- More than one-third of the informal caregivers in this sample reported depressive symptoms, with a higher prevalence among women than men.
- Perceived social support and physical activity frequency were independently associated with depressive symptoms.

**What are the implications of the main findings?**

- Poor perceived social support combined with physical inactivity was associated with the highest probability of depressive symptoms.

**Abstract:**

Background/Objectives: Informal caregivers are at an increased risk of developing depressive symptoms; however, the combined role of sex, perceived social support (PSS), and physical activity (PA) in this population remains insufficiently characterized. This study aimed to describe the prevalence of depressive symptoms among informal caregivers in a national health survey sample and to identify the biopsychosocial factors independently associated with them. Methods: This cross-sectional study used data from the Spanish National Health Survey 2023 (ESdE 2023). Informal caregivers (*n* = 2072) were identified using the caregiving status item. Depressive symptoms were assessed using the Patient Health Questionnaire-8 (PHQ-8; score ≥5), a screening measure that does not establish a clinical diagnosis of depression. Backward Wald logistic regression identified the independently associated factors, and predicted probabilities were estimated using PA frequency (PAF) and PSS, stratified by sex. Survey weights and complex sample procedures were not applied. Results: Depressive symptoms were reported by 34.5% of caregivers, and were higher among women than men (37.2% vs. 30.7%). Being female (OR = 1.34) and never or occasionally engaging in PA (OR = 1.82 and 1.54, respectively; reference: very frequently) were associated with higher odds of depressive symptoms, whereas each one-point increase in PSS was associated with lower odds (OR = 0.79). The predicted probabilities were highest among caregivers with poor PSS and no PA and lowest among those with strong PSS and frequent PA. Conclusions: Female sex, lower PSS, and infrequent PA were independently associated with depressive symptoms in this sample of informal caregivers.

## 1. Introduction

Informal caregivers typically provide unpaid support outside a formal or professional care framework to people with chronic conditions, disabilities, or other long-term care needs [1,2]. Informal caregiving may involve support with basic and instrumental activities of daily living as well as emotional and social support, often requiring sustained involvement over time [3]. As aging populations and chronic care needs increase, the demand for long-term care increases, and health and social care systems face growing pressure to ensure sustainable provision [4]. In this context, informal caregivers constitute a central component of care delivery, although increasing reliance on unpaid care raises concerns about the capacity of formal systems to meet growing needs without placing disproportionate demands on caregivers [5].

The consequences of informal caregiving may extend across multiple domains of caregivers’ lives, including physical health, psychological well-being, social participation, and occupational and financial circumstances [6]. Regarding physical health, caregiving has been associated with a higher incidence of severe stress, musculoskeletal conditions, and pain than non-caregiving, although evidence on caregivers’ overall mortality risk remains mixed [7]. Emotionally and psychologically, caregivers experiencing financial hardship and unmet social needs report higher rates of poor mental health, stress, and depression [8]. Socially, informal caregiving has been linked to increased loneliness and reduced social participation, largely because of diminished life satisfaction, restricted leisure time, and fewer opportunities for social engagement [9]. Finally, caregiving responsibilities may interfere with paid employment through reduced workforce participation, fewer working hours, or labor market withdrawal [10].

Among the biopsychosocial factors associated with depression in informal caregivers, sex, perceived social support (PSS), and physical activity (PA) have received considerable empirical support [11,12]. Female caregivers have consistently been found to be at greater risk of depression than their male counterparts, a finding reported in a meta-analysis of caregiver gender differences [13]. This disparity has been partly linked to differences in social support, as female caregivers have been found to receive less social support than men [14]. PSS is, in turn, among the most robust psychosocial correlates of depressive symptoms among informal caregivers and the general population [15,16]. Consistent with this association, caregiving has been shown to erode caregivers’ social networks over time, a mechanism through which reduced PSS may contribute to poorer mental health outcomes [17]. Similarly, PA has been inversely associated with depression risk in the general population [18] and among informal caregivers [11,19]; meta-analytic evidence also suggests benefits of PA interventions for caregivers’ mental health [20]. Greater PA and stronger PSS are also associated with lower psychological distress among adults with depression [21]. Within the biopsychosocial model, these factors can be situated in different dimensions: sex may capture biological differences and gendered sociocultural differences in caregiving roles; PSS is a psychosocial resource that, according to the stress-buffering hypothesis, may protect against the adverse effects of stressful circumstances [22]; and PA is a behavioral factor that may relate to depressive symptoms through psychosocial pathways such as self-esteem and self-efficacy [23].

However, these factors have mostly been examined separately among caregivers. Studies on Spanish caregivers have analyzed PA in relation to depression, anxiety, and mental health [11,19], and perceived social support in relation to mental health among caregivers aged 65 years or older [24], but, to our knowledge, they have not examined sex, PSS, and PA frequency together in relation to depressive symptoms. Given that both social support and PA differ by sex among caregivers [14,19], their simultaneous examination is needed to ascertain whether each remains independently associated with depressive symptoms.

This study aimed to examine depressive symptoms among informal caregivers in a national health survey sample from Spain. Specifically, the objectives were to (1) describe the prevalence of depressive symptoms and the biopsychosocial characteristics of informal caregivers by sex; (2) identify the biopsychosocial factors independently associated with depressive symptoms; and (3) estimate the combined association of physical activity frequency (PAF) and PSS with the probability of depressive symptoms by sex. The working hypotheses, informed by the existing literature, were that depressive symptoms would be more prevalent among women and that female sex, lower PSS, and lower PAF would be independently associated with depressive symptoms.

## 2. Materials and Methods

### 2.1. Design, Ethical Considerations, and Use of Artificial Intelligence

This cross-sectional study was based on data from the Spanish National Health Survey 2023 (ESdE 2023, available at: https://www.sanidad.gob.es/estadEstudios/estadisticas/encuestaSaludEspana/home.htm, accessed on 23 June 2026), a joint statistical operation of the Spanish National Statistics Institute (INE) and the Ministry of Health, conducted among the population residing in Spain aged ≥15 years.

As this study involved a secondary analysis of anonymized, publicly available microdata released by the INE and the Ministry of Health under Spain’s statistical secrecy framework, approval by a research ethics committee was not required.

During the preparation of this manuscript, the authors used AI tools to assist with the English translation and language editing. These tools were not used for study design, data analysis, interpretation of results, or any other scientific decision-making; all such decisions were made exclusively by the authors, who reviewed and edited all translated or AI-assisted content and take full responsibility for it.

### 2.2. Participants and Procedures

The ESdE is a health survey conducted jointly by the Spanish National Statistics Institute (INE) and the Ministry of Health to assess the health status of the population residing in Spain [25,26]. Conducted every three years, it collects information on health determinants, medication use, and healthcare service use, among other topics. The ESdE 2023 was conducted between August 2023 and August 2024 using a three-stage stratified random sampling design targeting the population aged ≥ 15 years residing in households in Spain. Data collection combined computer-assisted web interviews (CAWI) and computer-assisted personal interviews (CAPI). In the latter case, selected participants received prior notification of their selection, were invited to participate voluntarily, and were scheduled for an interview conducted by INE-trained and accredited interviewers.

Following sample selection, data collection, and data processing, the INE and Ministry of Health released fully anonymized microdata (available at: https://www.sanidad.gob.es/estadEstudios/estadisticas/microdatos/EncSalEsp/ESdEadulto_2023.zip; data accessed on 23 June 2026). The database contained responses from 21,032 participants, which corresponded to the initial sample for this study. Full details of the ESdE 2023 methodology, including complete questionnaires, sample size calculation, data processing, missing data handling, and other methodological characteristics, are publicly available on the Ministry of Health website [27].

As this study focused on the prevalence of depressive symptoms among informal caregivers, the following selection criteria were applied: (1) an affirmative response to item T.1: “Do you care, at least once a week, for an older person or someone with a chronic condition? Do not consider this if it is part of your job, “ and (2) valid responses to the depression severity item.

**Figure 1:**
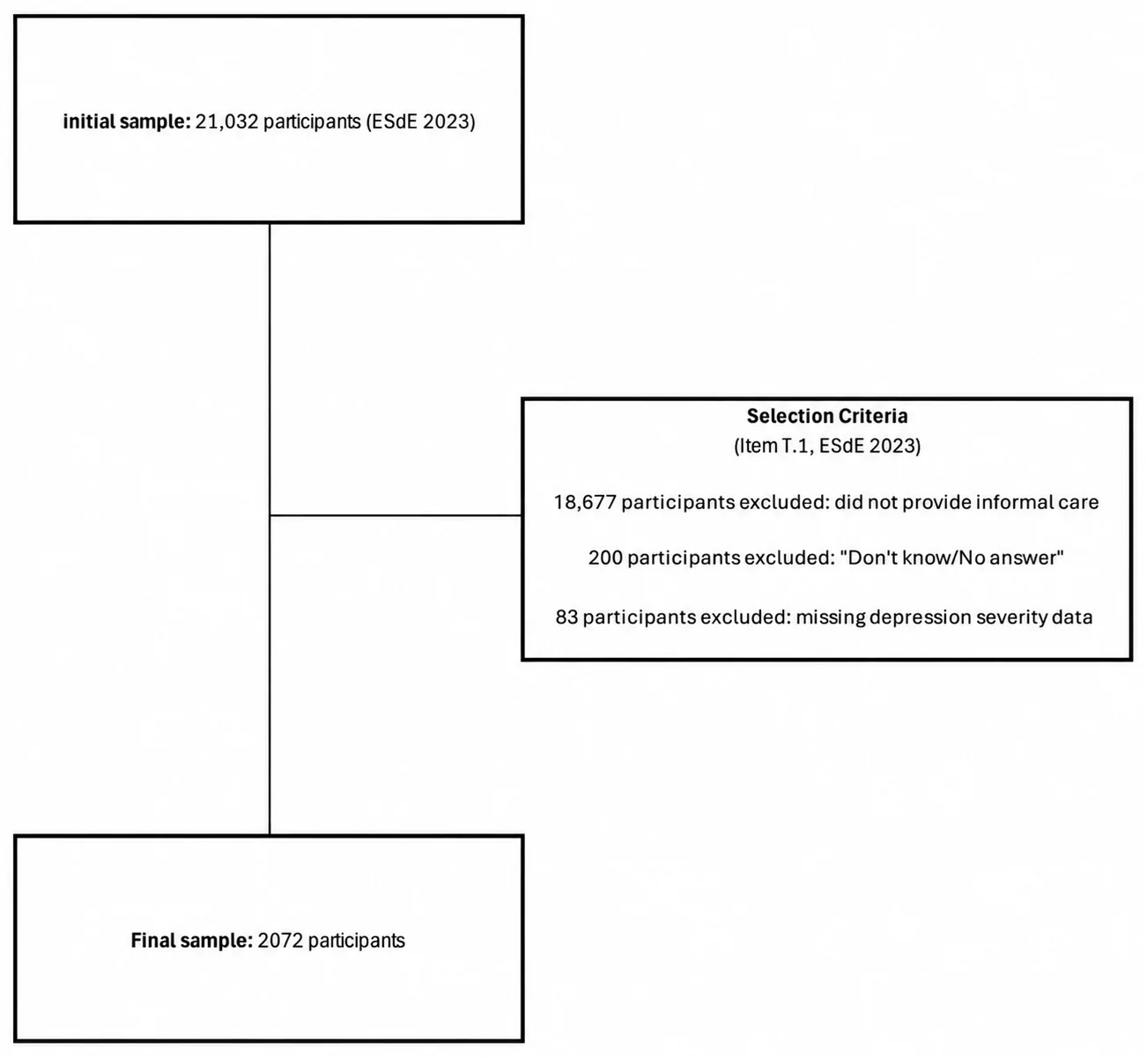
Flowchart of participant selection.

After applying these criteria, 18,677 participants who reported not caring for older or chronically ill people (i.e., who answered “no” to item T.1) were excluded. An additional 200 participants were excluded for responding “don’t know/no answer” to this item, and 83 participants were excluded for not having valid data on Depression Severity. Therefore, the final sample comprised 2072 participants (Figure 1).

### 2.3. Variables

#### 2.3.1. Outcomes

Depressive symptoms: A dichotomous variable derived from the *Depression Severity* item of the ESdE 2023, constructed from participants’ responses to the eight-item Patient Health Questionnaire (PHQ-8) [28]. The PHQ-8 yields a total score ranging from 0 to 24, allowing the severity of depressive symptoms to be classified from none (0–4 points) to severe (20–24). The ESdE 2023 reported PHQ-8 results by grouping participants according to depressive symptom severity as follows: none (0–4 points), mild (5–9), moderate (10–14), moderately severe (15–19), and severe (20–24). For the present study, participants were classified into two groups based on a cut-off score of 5: asymptomatic or no depressive symptoms, “No” (scores of 0–4), and symptomatic or presence of depressive symptoms, “Yes” (scores 5–24). This cut-off threshold was selected to distinguish caregivers exhibiting any level of depressive symptoms (ranging from mild to severe) from those who were asymptomatic. This threshold was not intended to establish a formal clinical diagnosis of major depressive disorder but rather served as a screening cutoff to identify self-reported depressive symptoms requiring screening or early support in informal caregiving populations. However, this dichotomous classification does not allow for an analysis of the specific degrees of clinical severity.

#### 2.3.2. Biopsychosocial Factors

Participant information included sex (self-reported by the participant during the survey interview. Options: Male or Female), age (in years), relationship to the care recipient (family member or non-family member), weekly time devoted to caregiving (less than 10 h, 10–20 h, or more than 20 h), educational level (primary, secondary, vocational, high school, or university education), social class (I–VI, Table S1), and body mass index (BMI) category (underweight, normal weight, overweight, or obesity).

Perceived Social Support (PSS) was assessed using participants’ responses to items S.1, S.2, and S.3 of the ESdE 2023, which correspond to the three-item Oslo Social Support Scale (OSSS-3) [29]. The OSSS-3 provides a sum score ranging from 3 to 14, with higher scores indicating stronger social support. In the present study, the OSSS-3 was evaluated as both a continuous score and a categorical variable. The continuous variable comprised the participants’ total sum scores on the scale. Furthermore, in line with the scoring conventions established in the original OSSS-3 instrument, which is widely used in European epidemiological studies, the categorical variable classified social support into three levels: poor (scores of 3–8), moderate (scores of 9–11), and strong support (scores of 12–14).

Physical activity frequency (PAF) was used to classify participants according to the frequency of leisure-time PA reported in item O.2 of the ESdE 2023: “Which of the following best describes how frequently you engage in PA during your leisure time?” The response options were as follows: I spend my leisure time almost entirely in sedentary activities, such as reading, watching television, or going to the cinema”; “I occasionally engage in physical or sporting activities, such as walking, cycling, gardening, light exercise, or recreational activities requiring slight physical effort”; “I engage in PA several times a month, such as sports, exercise, running, swimming, cycling, or team games”; and “I undertake sports or physical training several times a week” [27]. For the present study, these categories were labelled as never, occasionally, frequently, and very frequently, respectively.

### 2.4. Statistical Analyses

The distribution of continuous variables was assessed using the Kolmogorov–Smirnov test. Descriptive analyses were conducted for all the variables. Continuous variables are reported as medians and interquartile ranges (IQRs), whereas categorical variables are summarised using absolute and relative frequencies. The analyses were stratified by sex. Differences between men and women were examined using the Mann–Whitney U test for continuous variables and Pearson’s chi-square test for categorical variables.

A multivariable binary logistic regression model using the backward Wald elimination method (entry criterion: *p* < 0.05; removal: *p* > 0.10) was fitted to examine the biopsychosocial factors associated with depressive symptoms. Depressive symptoms (symptomatic vs. asymptomatic) were entered as the dependent variable, and sex, age, BMI category, OSSS-3 score, PAF, weekly caregiving time, relationship with the care recipient, social class, and educational level were entered as predictors. Item-specific missingness was low across candidate variables (OSSS-3: n = 45; BMI: n = 56; PAF: n = 16; educational level: n = 80; social class: n = 62; caregiving duration: n = 46) and addressed using complete-case analysis, resulting in a final analytical sample of N = 1805 caregivers. With n = 591 outcome events (presence of depressive symptoms) and k = 21 candidate predictor parameters in Step 1, the events-per-variable ratio was EPV = 28.1, well above the recommended threshold of 10. A sensitivity analysis using a fully adjusted model, with simultaneous entry (ENTER method) of all prespecified covariates, was conducted to confirm that the primary adjusted odds ratios followed the identical trends and magnitudes to those in the parsimonious backward Wald model, indicating that the results did not depend on the variable selection procedure. Model fit was assessed using the Hosmer–Lemeshow test, while overall calibration accuracy was evaluated using the Brier score. Model discrimination was supported by Nagelkerke’s R^2^. Influential cases were assessed using Cook’s distance, and multicollinearity was examined using tolerance and variance inflation factor (VIF) values. The results are expressed as odds ratios (ORs) with 95% confidence intervals (95% CIs).

All primary analyses were performed on unweighted data from the identified caregiver subsample. Because official survey expansion weights are calibrated to match the general national population rather than specific target subgroups (such as informal caregivers), applying general sampling weights within restricted subsamples can introduce extreme weight variability and destabilize multivariable models. Unweighted multivariable regression was therefore used to ensure a stable estimation of adjusted associations.

Finally, the predicted probabilities of depressive symptoms were calculated from the final additive multivariable model, which retained sex, PAF, and OSSS-3 scores, and were stratified by sex, which did not include formal multiplicative interaction terms. A forest plot was produced for the multiple binary logistic regression model, and separate heatmaps were generated for men and women to represent the predicted probabilities according to the PAF and categorical OSSS-3.

Statistical significance was set at *p* < 0.05. Analyses were performed using IBM SPSS Statistics version 27.0 (IBM Corp., Armonk, NY, USA), and graphical representations were generated using Python version 3.9.3.

## 3. Results

### 3.1. Normality Tests

The Kolmogorov–Smirnov test indicated that the continuous variables, age and OSSS-3, were not normally distributed in either the total sample or the sex-stratified subsamples (*p* < 0.001).

### 3.2. Descriptive Analysis and Differences by Sex

The final sample comprised 2072 informal caregivers, of whom 867 were men and 1205 were women. Their main sociodemographic, caregiving, and health-related characteristics are presented in Table 1.

The median age of the participants was 56 years (IQR = 16), with no significant differences between sexes (*Z* = −1.3, *p* = 0.201). The median OSSS-3 score was 12 (IQR = 3). Although the median was the same in both groups, women had significantly lower OSSS-3 scores than men (*Z* = −3.0, *p* = 0.003).

Among the categorical variables, significant associations with sex were observed for the relationship with the care recipient (χ^2^ = 6.7, *p* = 0.010), weekly caregiving time (χ^2^ = 6.9, *p* = 0.031), depressive symptoms (χ^2^ = 9.4, *p* = 0.002), PAF (χ^2^ = 10.6, *p* = 0.014), BMI category (χ^2^ = 37.9, *p* < 0.001), educational level (χ^2^ = 16.4, *p* = 0.002), and social class (χ^2^ = 16.8, *p* = 0.005).

In both sexes, the care recipient was predominantly a family member, with a higher proportion of men than women (97.3% vs. 95.1%, respectively). Women more frequently reported providing > 20 h of care per week (43.3% vs. 37.7%). The prevalence of depressive symptoms was also higher among women than among men (37.2% vs. 30.7%), as was the proportion of those who did not engage in leisure-time PA (27.0% vs. 21.8%). However, despite the statistically significant differences, the effect sizes for all these findings were small (Cramer’s *V* ranging from 0.057 to 0.137).

### 3.3. Multiple Binary Logistic Regression Model for Depressive Symptoms

**Figure 2:**
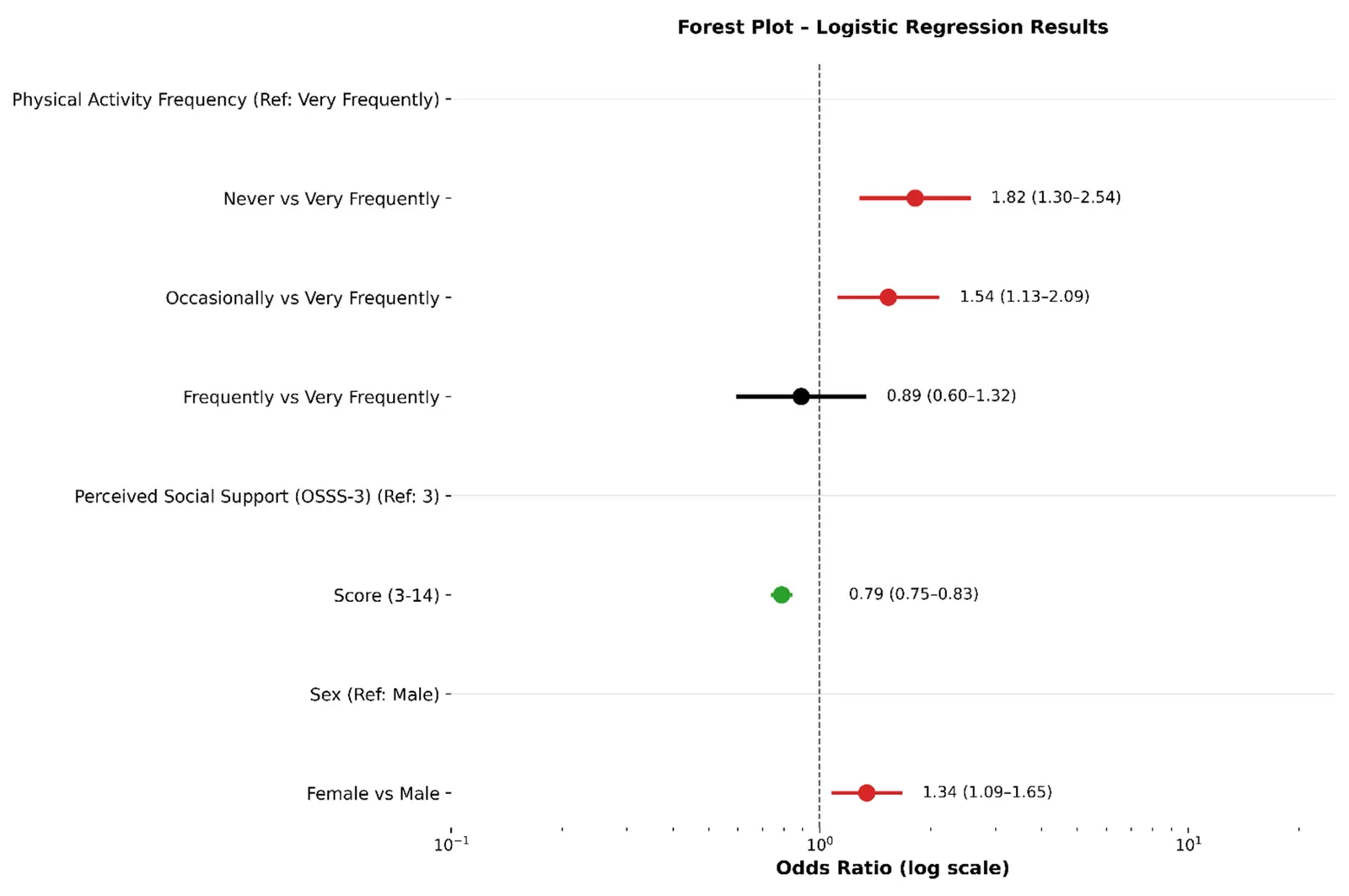
Forest plot of the multiple binary logistic regression model for depressive symptoms among informal caregivers (N = 1805; cases = 591). Perceived social support (OSSS-3) was evaluated as a continuous metric (OR expressed per 1-point increase in the score).

The multivariable logistic regression model was performed on a total of 1805 participants, including only those who provided valid data for all the covariates included, and comprising n = 591 cases with depressive symptoms (32.7%). The model showed an adequate overall fit according to the Hosmer–Lemeshow test (*p* = 0.770) and accounted for 8.5% of the variance in depressive symptoms (Nagelkerke *R*^2^ = 0.085) and displayed adequate calibration accuracy (Brier score = 0.205). No multicollinearity problems were detected (Tolerance values ranging from 0.62 to 0.98; VIF values ranging from 1.02 to 1.61) across candidate predictors, and no influential cases were identified (Cook’s *D* < 1). As shown in the forest plot (Figure 2), PAF, OSSS-3 score, and sex were retained as independent predictors of depressive symptoms.

Compared with the PAF Very Frequently category, caregivers in the PAF Never category had higher odds of depressive symptoms (OR = 1.82; 95% CI: 1.30–2.54), as did those in the PAF Occasionally category (OR = 1.54; 95% CI: 1.13–2.09). In contrast, higher OSSS-3 scores were associated with lower odds of depressive symptoms (OR: 0.79; 95% CI: 0.75–0.83). Women also had significantly higher odds of depressive symptoms than men (OR: 1.34; 95% CI: 1.09–1.65). The full model results are provided in Table S2. The sensitivity analysis, in which a fully adjusted model was fitted using the simultaneous variable entry (ENTER) method, yielded virtually identical results for all the main predictor variables, namely perceived social support (OSSS-3), frequency of physical activity (PAF), and sex, confirming the stability of the backward Wald parsimony model.

### 3.4. Predicted Probabilities

**Figure 3:**
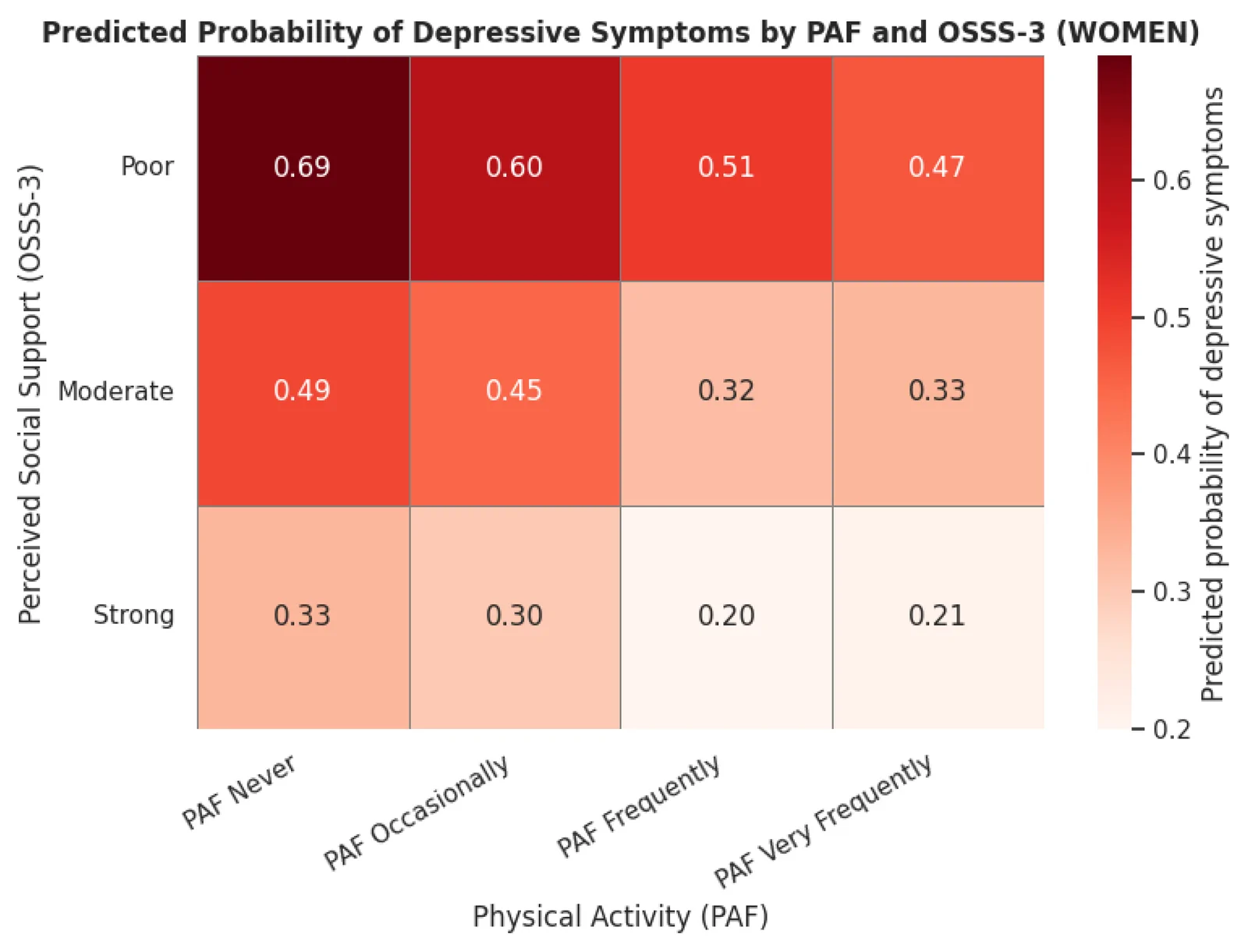

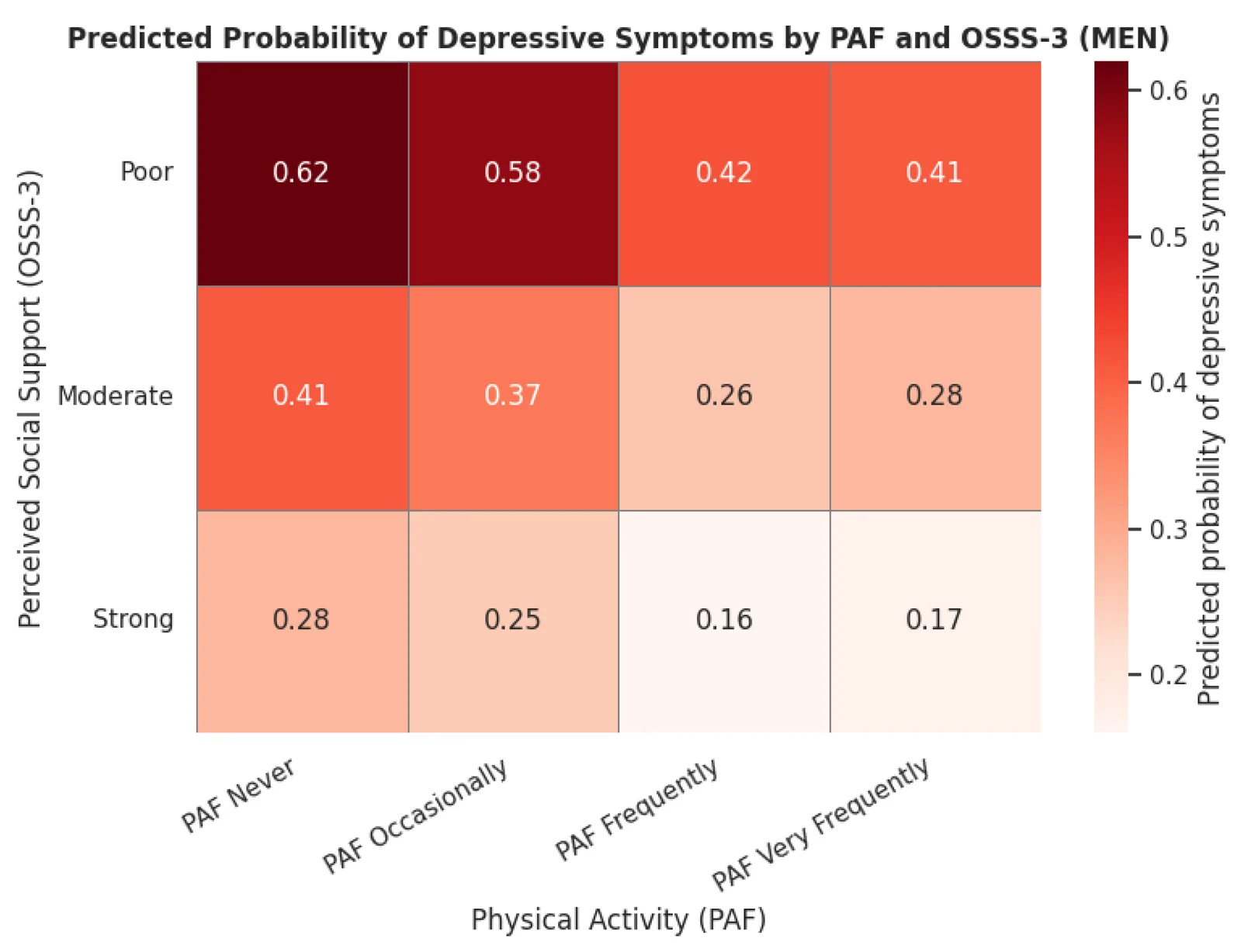
Predicted probabilities of depressive symptoms by physical activity (PAF) and perceived social support (OSSS-3), presented separately for women (**top**) and men (**bottom**). Perceived social support categories were defined based on OSSS-3 total scores: poor (3–8 points), moderate (9–11 points), and strong (12–14 points). Model predictions assume additive main effects; no interaction terms were tested.

Figure 3 shows the predicted probabilities of depressive symptoms for all combinations of the PAF and OSSS-3 scores, stratified by sex and age. A similar pattern was observed in both groups: the predicted probabilities were highest among caregivers with poor social support, particularly when combined with PAF Never. Conversely, the lowest predicted probabilities were observed among those with strong social support and frequent or very frequent participation in PA.

Among women, the highest-risk profile reached a predicted probability of 0.69 (95% C.I.: 0.66–0.74) for the combination of PAF Never and OSSS-3 Poor, whereas the lowest-risk profiles ranged from 0.20 (95% C.I.: 0.19–0.21) to 0.21 (95% C.I.: 0.21–0.22) for PAF Frequently or Very Frequently combined with OSSS-3 Strong. The same pattern was observed among men, with predicted probabilities ranging from 0.62 (95% C.I.: 0.56–0.65) for the highest-risk profile from 0.16 (95% C.I.: 0.15–0.16) to 0.17 (95% C.I.: 0.16–0.18) for the most favourable profiles. Visually, lower social support appeared to show a particularly pronounced descriptive separation across risk strata alongside activity frequency. The predicted probabilities were consistently higher in women than in men across all combinations, consistent with the independent association of sex observed in the regression model.

## 4. Discussion

This study examined depressive symptoms among informal caregivers participating in the 2023 Spanish National Health Survey from a biopsychosocial perspective, as specified by the variables available in this national survey. Specifically, it explored the prevalence of depressive symptoms and the characteristics of caregivers according to sex, identified the factors independently associated with depressive symptoms, and estimated the predicted probability of depressive symptoms across different combinations of PAF and PSS, stratified by sex. The findings showed that women had higher adjusted odds of depressive symptoms than men, whereas greater PSS and more frequent PA were independently associated with lower odds of depressive symptoms.

The provision of informal care in Spain has traditionally been strongly gendered, with women assuming primary responsibility for dependent relatives. Despite changing social attitudes, greater female participation in paid employment, and a gradual increase in men’s involvement in caregiving, the distribution of care remains inequitable in many countries. Previous studies have consistently reported a higher proportion of female caregivers than male caregivers, suggesting that recent changes have not yet resulted in a fully balanced allocation of caregiving responsibilities [30]. Women also constituted a larger proportion of caregivers in our sample, consistent with the predominance of women reported in the caregiving literature [3,30,31]. Although women represented a larger proportion of the sample, male and female caregivers were comparable in age; nevertheless, sex-related differences emerged in depressive symptoms.

The proportion of caregivers reporting depressive symptoms was significantly higher among women than among men (37.2% vs. 30.7%; χ^2^ = 9.4, *p* = 0.002). After adjustment, women had 34% higher odds of reporting depressive symptoms than men (OR: 1.34; 95% CI: 1.09–1.65). This pattern is in line with previous evidence indicating that depressive symptoms and other mental health difficulties are common among informal caregivers [1,32]. Our findings are also consistent with the meta-analysis reporting that female caregivers experience greater depressive symptoms and subjective burden than male caregivers [13]. In our sample, 43.3% of female caregivers reported providing more than 20 h of care, compared to 37.7% of male caregivers. However, weekly caregiving time was not retained in the final multivariable model. Previous studies have reported greater subjective burden, multifaceted strain, stress, and role overload among women [33,34]. Women have also been found to provide more hours of care, undertake a broader range of tasks, and assume greater responsibility for personal care, although these differences are generally small [13]. These factors, with the exception of caregiving hours, were not measured in our study, so they should be regarded as non-tested hypotheses that could help frame the sex difference rather than as findings.

Beyond the higher prevalence of depressive symptoms among women, they were more frequently represented among caregivers who never engaged in physical activity (27.0% vs. 21.8%). PSS and PAF were significant factors associated with depressive symptoms. The significance of PSS in our findings is consistent with the evidence from previous meta-analyses, which show that lower levels of PSS are associated with more severe depressive symptoms among informal caregivers [16]. This is particularly relevant because the OSSS-3 assesses caregivers’ perception of the availability and adequacy of their social resources [29].

Similarly, the less favorable PA profile observed among women and the independent association between PAF and depressive symptoms are consistent with previous observational studies involving Spanish informal caregivers [11,19] and meta-analytic evidence suggesting benefits for caregivers’ mental health and well-being [20]. However, a recent meta-analysis of randomized controlled trials among caregivers of people with Alzheimer’s disease and related dementias did not find a significant effect of PA interventions on depressive symptoms [35]. This contrast suggests that the association identified in our study should not be interpreted as evidence that PA directly reduces depressive symptoms, particularly given the cross-sectional design.

The predicted probabilities derived from the adjusted logistic regression model complemented these findings by showing how PSS and PAF were combined into more and less favorable profiles. Caregivers with poor social support who did not engage in PA had the highest estimated probability of depressive symptoms (69% in women and 62% in men), whereas those with strong social support who were active or very frequently active had the lowest probabilities (20–21% and 16–17%, respectively). Although the same pattern was observed in both sexes, women consistently showed higher estimated probabilities than men. However, in this study, the predicted probability profiles were descriptive visual representations derived from additive models, they did not constitute formal statistical evidence of interaction or effect modification, and should therefore be interpreted with caution.

The findings should be interpreted by considering the study design and measures used. (1) The cross-sectional nature of the data prevents the temporal direction of the observed associations from being established; reverse causality is plausible, as depressive symptoms may reduce PA [36] and alter the perception of available social support. (2) Depressive symptoms were identified using a screening measure dichotomized at a cut-off of 5 points, which groups mild to severe symptoms together and does not retain information on severity; therefore, estimates may differ with other thresholds. These symptoms should not be interpreted as a clinical diagnosis of depression, and the multivariable model explained a modest proportion of variance (Nagelkerke R^2^ = 8.5%), reflecting population-level associations rather than strong individual prediction. Furthermore, the modest effect sizes require caution when evaluating their practical clinical impact. (3) PA was self-reported, which may be subject to reporting bias, and was assessed only in terms of frequency, without considering its type, duration, or intensity; therefore, the findings cannot be interpreted in terms of a specific “dose” of PA, whereas the OSSS-3 provides a brief assessment of PSS without distinguishing its source, quality, or support received. (4) Sampling weights and complex survey design elements were not incorporated into the multivariable models due to the subgroup-specific focus on informal caregivers. While unweighted estimates provide valid internal measures of association in this sample, caution should be exercised when extrapolating absolute point-prevalence estimates directly to the national population of informal caregivers. (5) Residual confounding cannot be ruled out, as relevant caregiving-related factors, such as caregiver burden, the dependency level of the care recipient, employment status, or financial difficulties, were not included in the analysis. (6) Missing data were handled using complete-case analysis, which excluded 267 participants (12.9%) from the multivariable model; if data were not missing at random, the estimates could be biased.

Longitudinal studies are needed to clarify the temporal relationships between PSS, PA, and depressive symptoms among informal caregivers. Intervention studies are also required to determine whether improving PSS or PA frequency is followed by lower levels of depressive symptoms in the long term. Future research should also include more detailed measures of caregiving intensity, task complexity, subjective burden, PSS, PA, employment conditions, financial strain, self-care, and participation in meaningful activities. This would provide a more comprehensive understanding of the factors associated with depressive symptoms and allow for better control of residual confounding. It may also help examine whether these factors contribute to the differences observed between women and men in the study.

## 5. Conclusions

Depressive symptoms were present in more than one in three informal caregivers in this study subpopulation, with a higher prevalence among women than among men. The predicted probability of depressive symptoms was highest among caregivers with poor social support and physical inactivity and lowest among those with strong social support and frequent PA. Female sex, lower PSS, and infrequent PA were independently associated a higher likelihood of experiencing depressive symptoms; however, the low proportion of variance explained highlights that these modifiable factors account for only a small part of a highly complex and multifactorial condition, which is determined by factors related to clinical care and support that were not measured. PSS and PA frequency, as potentially modifiable correlates, warrant further investigation in longitudinal and interventional studies to determine their potential usefulness and practical relevance in supporting carers’ mental health.

## Figures and Tables

**Table 1:** Descriptive analysis.

Variables	Men = 867	Women = 1205	Total = 2072	Z	df	*p*	V
	Median (IQR)	Median (IQR)	Median (IQR)				
Age (Years)	56 (17)	56 (16)	56 (16)	−1.3	-	0.201	-
OSSS-3 (Score)	12 (3)	12 (2)	12 (3)	−3.0	-	0.003	-
Caregiver: Who?	Men = 867	Women = 1025	Total = 2072	X^2^	df	*p*	V
Family	844 (97.3)	1146 (95.1) *	1990 (96.0)	6.7	1	0.010	0.057
Non-Family	23 (2.7)	59 (4.9) *	82 (4.0)				
Caregiver: Hours?	Men = 848	Women = 1178	Total = 2026				
Fewer than 10	346 (40.8)	423 (35.9) *	769 (38.0)	6.9	2	0.031	0.058
Between 10 and 20	182 (21.5)	245 (20.8) *	427 (21.1)				
More than 20	320 (37.7)	510 (43.3) *	830 (41.0)				
Perceived Support	Men = 849	Women = 1178	Total = 2027				
Poor (Score: 3–8)	60 (7.1)	70 (5.9)	130 (6.4)	3.3	2	0.191	0.04
Moderate (Score: 9–11)	337 (39.7)	435 (36.9)	772 (38.1)				
Strong (Score: 12–14)	452 (53.2)	673 (57.1)	1125 (55.5)				
Depressive Symptoms	Men = 867	Women = 1025	Total = 2072				
Yes	266 (30.7)	448 (37.2) *	714 (34.5)	9.4	1	0.002	0.067
No	601 (69.3)	757 (62.8) *	1358 (65.5)				
Physical Activity	Men = 859	Women = 1197	Total = 2056				
Never	187 (21.8)	323 (27.0) *	510 (24.8)	10.6	3	0.014	0.072
Occasionally	387 (45.1)	543 (45.4)	930 (45.2)				
Frequently	132 (15.4)	152 (12.7)	284 (13.8)				
Very frequently	153 (17.9)	179 (15.0)	332 (16.1)				
BMI	Men = 852	Women = 1164	Total = 2016				
Underweight	6 (0.7)	27 (2.3) *	33 (1.6)	37.9	3	<0.001	0.137
Normal	285 (33.5)	515 (44.2) *	800 (39.7)				
Overweight	408 (47.9)	425 (36.5) *	833 (41.3)				
Obesity	153 (18.0)	197 (16.9)	350 (17.4)				
Education Level	Men = 843	Women = 1149	Total = 1992				
Primary	87 (10.3)	168 (14.6) *	255 (12.8)	16.4	4	0.002	0.091
Secondary	230 (27.3)	265 (23.1) *	495 (24.8)				
Vocational Training	174 (20.6)	246 (21.4)	420 (21.1)				
High School	132 (15.7)	139 (12.1) *	271 (13.6)				
University	220 (26.1)	331 (28.8)	551 (27.7)				
Social Class	Men = 859	Women = 1151	Total = 2010				
I	131 (15.3)	179 (15.6)	310 (15.4)	16.8	5	0.005	0.091
II	81 (9.4)	103 (8.9)	184 (9.2)				
III	181 (21.1)	250 (21.7)	431 (21.4)				
IV	140 (16.3)	129 (11.2) *	269 (13.4)				
V	246 (28.6)	336 (29.2)	582 (29.0)				
VI	80 (9.3)	154 (13.4) *	234 (11.6)				

n: participants; %: percentage; OSSS-3: Oslo Social Support Scale, score (3–14); Z: Z-statistic; df: degrees of freedom; *p*: *p*-value; *: Difference in proportions with *p* < 0.05 from pairwise z-test for independent proportions with Bonferroni correction or significant; X^2^: Chi-square; V: Cramer’s V; BMI: Body mass index, I–VI: I, the best social class, VI, the worst). Note: Values are expressed as median (IQR) or n (valid %). Percentages are calculated based on valid responses for each variable. Sample denominators for specific variables vary due to item-specific missing data: BMI (n = 2016; n = 56 missing [2.7%]), Educational Level (n = 1992; n = 80 missing [3.9%]), Social Class (n = 2010; n = 62 missing [3.0%]), Caregiving Duration (n = 2026; n = 46 missing [2.2%]), Perceived Social Support/OSSS-3 (n = 2027; n = 45 missing [2.2%]), and Physical Activity (n = 2056; n = 16 missing [0.8%]).

## Data Availability

Microdata are available in https://www.sanidad.gob.es/estadEstudios/estadisticas/microdatos/EncSalEsp/ESdEadulto_2023.zip (accessed on 12 October 2026).

## Supplementary Materials

The following supporting information can be downloaded at https://www.mdpi.com/article/10.3390/healthcare14203485/s1, Table S1: Description of social classes based on occupational occupation.; Table S2: Multiple binary logistic regression model for depressive symptoms.

## Abbreviations

The following abbreviations are used in this manuscript.

PA	Physical Activity
PAF	Physical Activity Frequency
PSS	Perceived Social Support
