# Supplementary material for: Sex, Perceived Social Support, and Physical Activity as Correlates of Depressive Symptoms in Informal Caregivers: A Cross-Sectional Analysis of the Spanish National Health Survey 2023
Source: Healthcare (Basel). 2026 Oct 16;14(20):3485. doi: 10.3390/healthcare14203485 (PMC13704005; doi:10.3390/healthcare14203485)
Supplement: Supplementary file 1 [file healthcare-14-03485-s001.zip › S1 Description of social classes based on occupational occupation.pdf]

Supplementary Table S1. Description of social classes based on occupational occupation.

**CLASS I**—Directors and managers of establishments with 10 or more employees, and professionals traditionally associated with university degrees.
**CLASS II**—Directors and managers of establishments with fewer than 10 employees, professionals traditionally associated with university degrees and other technical support professionals. Technical support professionals. Athletes and artists.
**CLASS III**—Intermediate occupations and self-employed workers.
**CLASS IV** - Supervisors and workers in skilled technical occupations.
**CLASS V**—Skilled workers in the primary sector and other semi-skilled workers. Semi-skilled workers.
**CLASS VI**—Unskilled workers.
