# Supplementary material for: Sex, Perceived Social Support, and Physical Activity as Correlates of Depressive Symptoms in Informal Caregivers: A Cross-Sectional Analysis of the Spanish National Health Survey 2023
Source: Healthcare (Basel). 2026 Oct 16;14(20):3485. doi: 10.3390/healthcare14203485 (PMC13704005; doi:10.3390/healthcare14203485)
Supplement: Supplementary file 1 [file healthcare-14-03485-s001.zip › S2 Multiple binary logistic regression model for depressive symptoms.pdf]

Table S2. Multiple binary logistic regression model for depressive symptoms.

	B	S.E.	Wald	Sig.	Exp(B)	95% C.I. for EXP(B)	
						Lower	Upper
Perceived Social Support (Score)	-0.237	0.028	73.078	<0.001	0.789	0.748	0.833
Sex (Ref. Men)	0.295	0.106	7.771	0.005	1.343	1.091	1.652
Physical Activity Frequency (Ref. Very frequently)			23.456	<0.001			
Never	0.597	0.171	12.170	<0.001	1.816	1.299	2.540
Occasionally	0.43	0.156	7.626	0.006	1.538	1.133	2.087
Frequently	-0.115	0.201	0.328	0.567	0.891	0.601	1.321
Constant	1.478	0.351	17.750	<0.001	4.383		

B (Beta); S.E. (Standard error); Sig. (Statistical significance); Exp (Exponential regression); C.I. (Confidence interval).
